# Identification of a novel *ipp* gene cluster responsible for 2-isopropylphenol degradation in strain *Rhodococcus* sp. D-6

**DOI:** 10.1128/aem.00995-25

**Published:** 2025-08-14

**Authors:** Qian Zhu, Kangning Wei, Kaihua Pan, Gang Hu, Weihao Zhu, Yanni Huang, Changchang Wang, Qian Li, Mingliang Zhang, Jiguo Qiu, Qing Hong

**Affiliations:** 1 Department of Microbiology, College of Life Sciences, Nanjing Agricultural University, Key Laboratory of Agricultural and Environmental Microbiology, Ministry of Agriculture and Rural Affairs 70578, Nanjing, Jiangsu, China; 2 Modern Agricultural Analysis and Testing Center, Nanjing Agricultural University 70578, Nanjing, Jiangsu, China; Danmarks Tekniske Universitet The Novo Nordisk Foundation Center for Biosustainability, Kgs. Lyngby, Denmark

**Keywords:** *Rhodococcus *sp. D-6, biodegradation, isoprocarb, 2-isopropylphenol monooxygenase IppA1A2, 2-isopropylhydroquinone dioxygenase IppB

## Abstract

Isoprocarb (IPC), a widely used carbamate insecticide, causes environmental contamination and poses risks to humans and ecosystems. *Rhodococcus* sp. D-6, capable of utilizing IPC as the sole growth substrate, was isolated by our lab. Strain D-6 initiates IPC degradation through ester bond hydrolysis by the hydrolase IpcH, yielding 2-isopropylphenol (IPP). In this study, the catabolic pathway of IPP in strain D-6 was elucidated, and the *ipp* gene cluster responsible for its degradation was predicted by transcriptomic analysis. Key genes, including a novel two-component IPP monooxygenase gene *ippA1A2* and a 2-isopropylhydroquinone dioxygenase gene *ippB*, are responsible for the successive conversion of IPP, resulting in the ring cleavage of IPP. IppA1, a group D flavin-dependent monooxygenase, along with its reductase component IppA2, hydroxylates IPP to 2-isopropylhydroquinone using reduced nicotinamide adenine dinucleotide (NADH) and flavin adenine dinucleotide (FAD) as cofactors. The optimal molar ratio of IppA1 to IppA2 was 3:1. The apparent *K*_m_ and *k*_cat_ values of IppA1 for IPP were 60.52 ± 1.27 µM and 70.07 ± 0.85 s^−1^, respectively. IppB, an extradiol dioxygenase, catalyzes 2-isopropylhydroquinone ring cleavage to generate 2-isopropyl-4-hydroxymuconic semialdehyde. The apparent *K*_m_ and *k*_cat_ values of IppB for 2-isopropylhydroquinone were 29.07 ± 1.54 μM and 147.65 ± 1.92 s^−1^, respectively. This study provides critical insights into the molecular mechanisms of IPP degradation, contributing to a comprehensive understanding of IPC degradation.

**IMPORTANCE:**

Carbamate insecticides kill pests by inhibiting the activity of acetylcholinesterase (AChE) and have been widely used in agriculture. Compared to the studies on the degradation mechanisms of carbofuran and carbaryl, little is known about IPC degradation. An IPC-degrading strain *Rhodococcus* sp. D-6 was isolated by our lab, and the hydrolase gene *ipcH* responsible for hydrolyzing IPC to IPP has been identified previously. This study further elucidates the IPP degradation pathway in strain D-6 and identifies the novel two-component IPP monooxygenase IppA1A2 and 2-isopropylhydroquinone dioxygenase IppB, which are respectively responsible for IPP hydroxylation and its subsequent ring cleavage. These findings enhance our understanding of the microbial degradation mechanism of IPC.

## INTRODUCTION

Carbamate insecticides are widely used in agriculture to control insects and nematode pests (1), killing them by inhibiting the activity of acetylcholinesterase (AChE) (2, 3). However, their excessive use has led to the accumulation of residues in the environment. Since AChE is also present in non-target organisms, including humans, these residues pose health risks (4–7). Because of this, it is important to investigate their microbial degradation.

At present, the degradation mechanism of bicyclic carbamate insecticides (such as carbofuran and carbaryl) has been intensively investigated; the pathways involved have been elucidated, and the involved genes have been identified (8–17). In comparison, research on the degradation mechanism of isoprocarb (IPC), a representative monocyclic carbamate insecticide, remains relatively scarce. To date, several IPC-degrading strains, including *Streptococcus thermophilus*, *Lactobacillus bulgaricus*, *Lactobacillus plantarum*, *Bacillus licheniformis* B-1, *Sphingobium* sp. R-7, *Acinetobacter* sp. ZX01*,* and *Rhodococcus* sp. D-6, have been isolated (18–22). Among them, strain D-6 isolated by our lab is the only strain capable of utilizing IPC as the sole growth substrate. It initiates its degradation through ester bond hydrolysis to generate 2-isopropylphenol (IPP).

Residual IPP has been detected in various environmental samples, including sediments from Wisconsin lake, Siberian crude oil, and in sport fish species such as walleye, pike, and yellow perch (23, 24). Additionally, studies have reported the toxicity of IPP on different organisms. Choi et al. revealed that IPP was toxic to *Vibrio fischeri*, the invertebrate *Ceriodaphnia dubia,* and the fish *Pimephales promelas* (25). IPP inhibits the germination and growth of the dicotyledons *Lactuca sativa* and monocotyledons *Allium cepa* (26) and disrupts the endocrine systems of wildlife and humans by interacting with hormone receptors (27). Moreover, IPP exhibits toxicity toward the gram-negative strain *Escherichia coli* DH5α, gram-positive strain *Bacillus cereus* 168, yeast strain *Pichia pastoris* GS115, and algae *Chlorella ellipsoidea* (22). Notably, the toxicity of IPP toward these organisms was found to be higher compared to its parent compound, IPC (22). Investigations into IPP degradation can be traced back to 1994 when Reichlin et al. investigated it in *Pseudomonas* sp. HBP1 Prp (28). Later, in 2010, Toyama et al. isolated *Pseudomonas* sp. MS-1, another strain capable of degrading IPP (29). However, the genes responsible for IPP metabolism have not been identified. Consequently, investigation of IPP degradation is of great significance for mitigating its environmental impact and elucidating the molecular mechanisms involved.

In our previous study, an IPC-degrading strain *Rhodococcus* sp. D-6 was isolated, and the hydrolase gene *ipcH* that initiates IPC degradation by hydrolyzing IPC to IPP was identified (22). The present study further investigated the IPP degradation pathway, identifying the *ipp* gene cluster responsible for IPP catabolism.

## RESULTS

### Prediction of IPP degradation-related genes through transcriptome analysis

As previous studies revealed that IPP degradation was induced by IPP in strain D-6 (22), transcriptome analysis was conducted to identify the genes involved in IPP degradation. In total, 16,090,993 and 15,938,567 high-quality reads were obtained from strain D-6 cultured with glucose and IPP as the sole growth substrates, respectively (Table S1). Among these, 14,739,488 and 15,279,075 reads were genome-mapped reads in glucose and IPP treatments, respectively (Table S1). The number of genes expressed in the glucose and IPP treatments was 5,097 and 5,101, respectively (Tables S5 and S6. Between the treatments, a total of 4,183 genes were identified as being differentially expressed, with a log_2_ fold change of >1 or <−1 (reads/kb per million reads) (Table S7). Among these, the transcription of 3,735 genes was upregulated and the transcription of 448 genes was downregulated in the presence of IPP (Tables S8 and S9).

From transcriptome analysis and functional annotation of differentially expressed genes, a 9 kb gene cluster (named *ipp* gene cluster) comprizing eight genes (*orf2559* to *orf2566*) was predicted to be involved in IPP degradation (Tables S1 and S10). All genes within this cluster were upregulated >2^2^-fold in the presence of IPP (Table 1). Among the upregulated genes, *orf2559* (4.46 log_2_ fold change, named *ippA1*) and *orf2561* (3.93 log_2_ fold change, named *ippA2*) were predicted to encode a two-component flavin-dependent monooxygenase system involved in IPP hydroxylation. The *ippA1* gene encodes the oxygenase of this system, exhibiting the highest sequence identity of 31.51% with the 3-hydroxy-9,10-secoandrosta-1,3,5(10)-triene-9,17-dione 4-hydroxylase oxygenase component HsaA from *Rhodococcus jostii* RHA1 (30). The *ippA2* gene encodes the reductase of this system, exhibiting the sequence identity of 31.29% with the 3-hydroxy-9,10-secoandrosta-1,3,5(10)-triene-9,17-dione 4-hydroxylase reductase component HsaB from *R. jostii* RHA1 (30). Notably, HsaAB, encoding two-component flavin-dependent monooxygenases, catalyzes the hydroxylation of 3-hydroxy-9,10-secoandrosta-1,3,5(10)-triene-9,17-dione (30). HsaA is the oxygenase component and HsaB is the reductase component (30). From this, *ippA1* and *ippA2* were hypothesized to encode a monoconfirmed oxygenase system catalyzing the hydroxylation of IPP, which was further confirmed through experimental validation.

**TABLE 1:** Deduced functions of open reading frames (ORFs) within the *ipp* gene cluster

Gene	Product size (no. of amino acid)	Proposed product	Homologous protein in UniProtKB/Swiss-Prot (GenBank accession no.)	Identity (%)	Log_2_ fold change	*P* values
*orf2559* (*ippA1*)	388	IPP monooxygenase, oxygenase component	3-hydroxy-9,10-secoandrosta-1,3,5(10)-triene-9,17-dione 4-hydroxylase, oxygenase component HsaA (Q0S811)	32	4.46	9.22 × 10^−25^
*orf2560*	214	Synthase	3,4-Dihydroxy-2-butanone 4-phosphate synthase RibBA (A4X639)	42	4.46	2.70 × 10^−10^
*orf2561* (*ippA2*)	162	IPP monooxygenase, reductase component	4-nitrophenol 4-monooxygenase/4-nitrocatechol 2-monooxygenase, reductase component NpcB (Q6F4M9)	36	3.93	1.97 × 10^−13^
*orf2562*	421	Hypothetical protein	None	None	2.03	1.12 × 10^−5^
*orf2563* (*ippB*)	211	2-Isopropylhydroquinone dioxygenase	Biphenyl-2,3-diol 1,2-dioxygenase BphC2 (P47232)	84	5.95	1.80 × 10^−8^
*orf2564*	296	Hypothetical protein	Fumarylacetoacetate hydrolase domain-containing protein 2 homolog (Q54BF3)	44	3.98	3.25 × 10^−12^
*orf2565*	663	Synthetase	Acetoacetyl-CoA synthetase AcsA2 (Q9Z3R3)	42	3.28	8.17 × 10^−14^
*orf2566*	209	Hypothetical protein	Putative NADH dehydrogenase/NAD(P)H nitroreductase SCO7141 (Q9FBV0)	57	2.84	8.02 × 10^−13^

### IppA1A2 catalyzes IPP hydroxylation to generate 2-isopropylhydroquinone

#### Expression and purification of IppA1 and IppA2

To identify the function of *ippA1* and *ippA2*, they were individually expressed in *E. coli* BL21 (DE3) and subsequently purified by Ni-affinity chromatography. The purified IppA1 and IppA2 appeared as a single band on SDS-PAGE, exhibiting the molecular mass of 42.0 and 20.9 kDa, respectively, consistent with their theoretical mass (Fig. S1A and S1B).

#### Characterization of the reductase IppA2

As the reductase component of IPP monooxygenase, the NAD(P)H-oxidizing activity of IppA2 was determined in the presence of FAD or FMN. IppA2 exhibited oxidizing activity toward NADH in the presence of FAD (Fig. S2 A), while no activity was observed for NADH with FMN (Fig. S2 B). Additionally, IppA2 showed no oxidizing activity toward NADPH with either FAD (Fig. S2 C) or FMN (Fig. S2 D). The apparent *k*_cat_/*K*_m_ values of IppA2 toward NADH when FAD was used as a second substrate and FAD when NADH was used as a second substrate were 0.50 and 11.17 s^−1^ µM^−1^, respectively (Fig. S3). The kinetic assays revealed apparent *K*_m_ values of IppA2 toward NADH and FAD of 20.73 ± 2.45 and 2.42 ± 0.32 µM^−1^, respectively (Fig. S3). These results indicated that the reductase component IppA2 utilized NADH to reduce FAD by producing FADH_2_.

#### Characterization of the IPP monooxygenase IppA1A2

**Fig 1:**
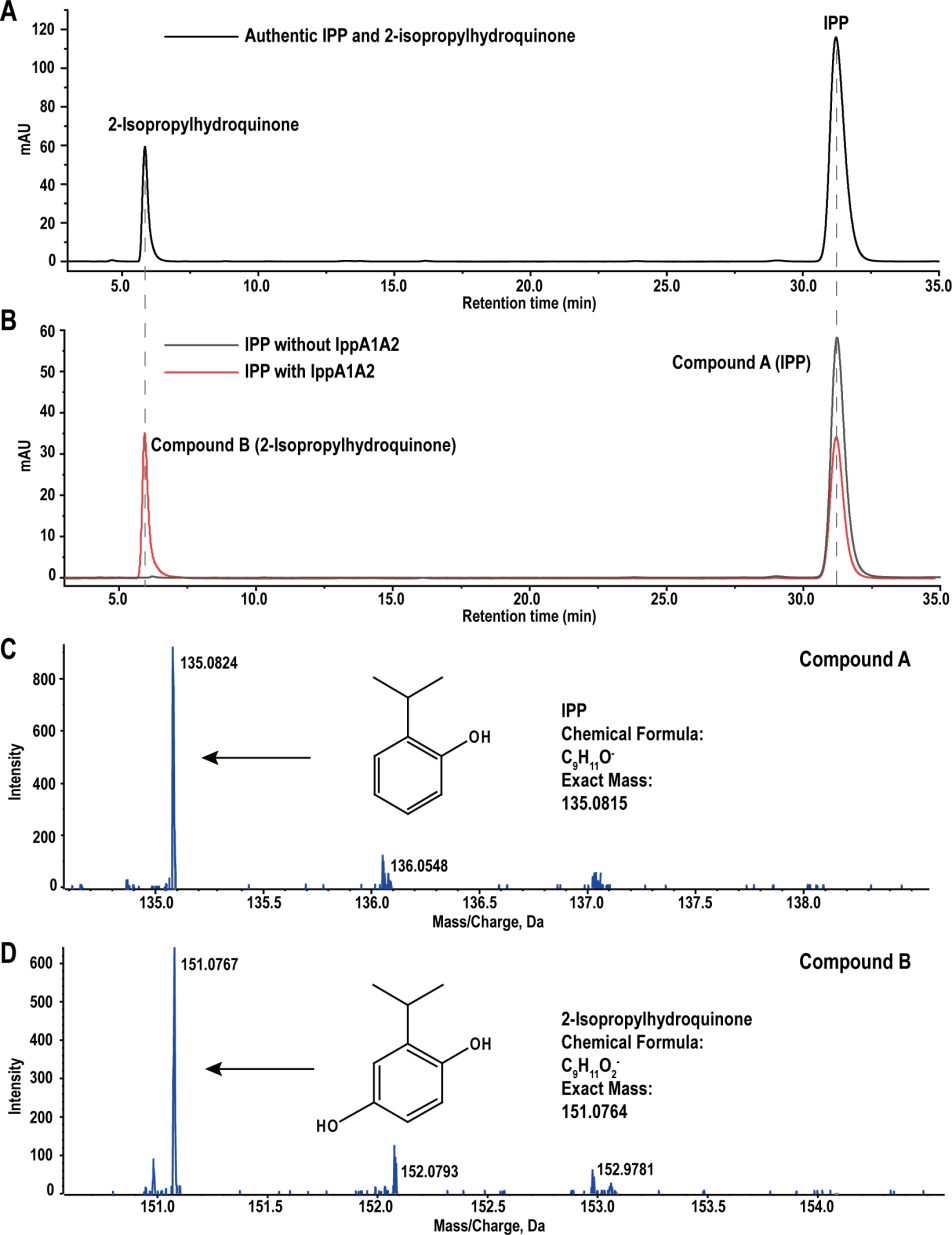
Identification of hydroxylated product of IPP by IppA1A2. (**A**) HPLC analysis of authentic IPP and 2-isopropylhydroquinone. (**B**) The metabolites that appeared during the conversion of IPP by IppA1A2. (**C**) MS analysis of compound A (*m/z* 135.0824 [M-H]^−^), which was identified as IPP (C_9_H_11_O^−^, with *m/z* 135.0815) with a 4.1 ppm error. (**D**) MS analysis of compound B (*m/z* 151.0767 [M-H]^−^), which was identified as 2-isopropylhydroquinone (C_9_H_11_O_2_^−^, *m/z* 151.0764) with a 1.5 ppm error.

IppA1 could not hydroxylate IPP independently; it catalyzes the hydroxylation of IPP in the presence of IppA2 (Fig. S4 A).Fig. S4A). The optimal molar ratio of IppA1 to IppA2 was determined to be 3:1 (Fig. S4 A). Under optimal conditions, the apparent *K*_m_, *k*_cat_ and *k*_cat_/*K*_m_ of IppA1 for IPP were 60.52 ± 1.27 µM, 70.07 ± 0.85 s^−1^, and 1.16 s^−1^ µM^−1^, respectively (Fig. S4 B). Two compounds were identified in the IppA1A2 enzymatic reaction system. Compound A, with a retention time of 31.23 min, corresponded to the retention time of authentic IPP (Fig. 1A and B). Compound B, with a retention time of 6.05 min, matched the retention time of the authentic 2-isopropylhydroquinone (Fig. 1A and B). The predominant deprotonated molecular ion of compound A was *m/z* 135.0824 [M-H]^−^. Based on molecular ion mass analysis, compound A was identified as IPP (C_9_H_11_O^−^, with *m/z* 135.0815), with a 4.1 ppm error (Fig. 1C). The molecular ion peak [M-H]^−^ of compound B was *m/z* 151.0767, aligning with the deprotonated derivative of 2-isopropylhydroquinone (C_9_H_11_O_2_^−^, *m/z* 151.0764) with a 1.5 ppm error (Fig. 1D). These results indicated that IppA1A2 hydroxylated IPP to 2-isopropylhydroquinone.

**Fig 2:**
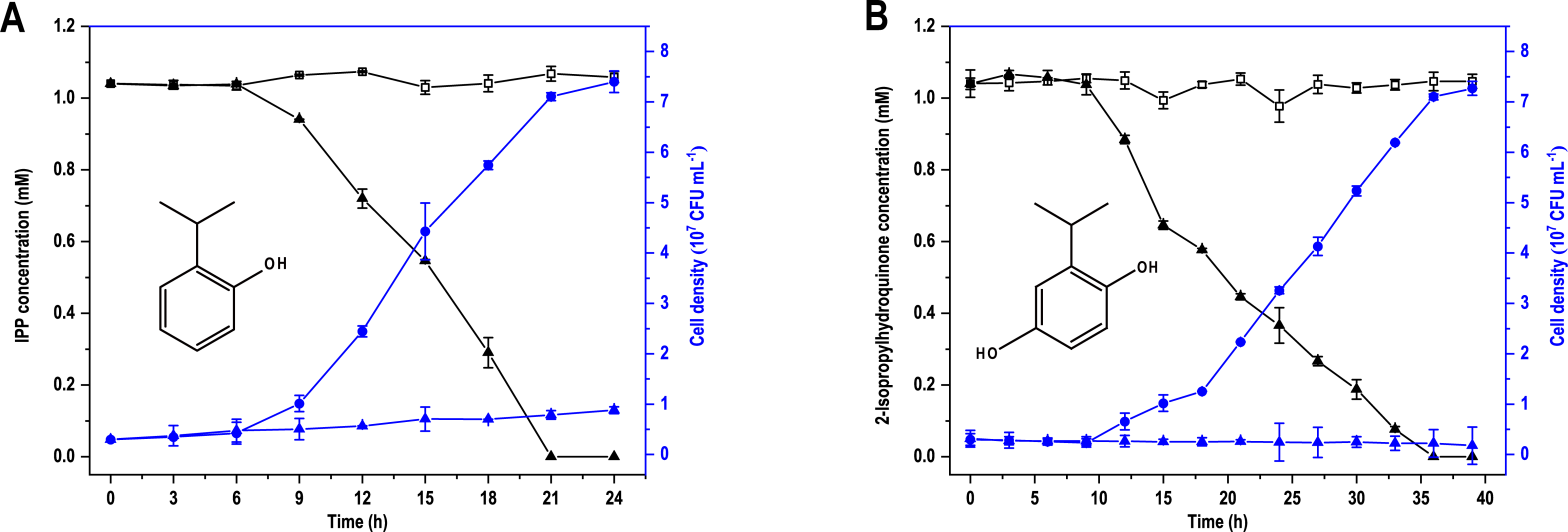
Degradation and growth of strain D-6 on IPP (**A**) and 2-isopropylhydroquinone (**B**). (A) and (B) depict the molecular structural formula of IPP and 2-isopropylhydroquinone, respectively. White square represents the substrate control; black triangle represents the substrate with strain D-6; blue circle represents the cell density of strain D-6 with the substrate; and blue triangle represents the cell density of strain D-6 without the substrate. Cell growth was determined by the colony counting method. Error bars represent the standard errors from triplicates.

In a previous study, it was speculated that the hydroxylation product of IPP in strain D-6 was 3-isopropylcatechol based on its ability to degrade this compound and the degradation pathway of IPP in *Pseudomonas* sp. HBP1 Prp (22, 28). To further determine the product of IPP hydroxylation, additional biodegradation experiments were carried out. Strain D-6 was capable of utilizing IPC as the sole growth substrate (22). The initial degradation step of IPC was its hydrolysis to methylcarbamic acid and IPP, with the former spontaneously hydrolyzing to methylamine and CO_2_. In the present study, strain D-6 was unable to grow on methylcarbamic acid and methylamine as sole growth substrates (data not shown). We therefore hypothesized that strain D-6 grows on downstream metabolites of IPC, including IPP and its hydroxylated derivatives. Strain D-6 could completely degrade 1.04 mM IPP within 21 h (Fig. 2A). The cell density of strain D-6 increased 24.7-fold from 3.0 × 10^6^ to 7.4 × 10^7^ CFU mL^−1^. However, in MSM without IPP, the cell density of strain D-6 showed no significant change (Fig. 2A). These results suggested that strain D-6 could grow solely on IPP.

IPP hydroxylation has the potential to yield four isomeric products: 2-isopropylhydroquinone, 3-isopropylcatechol, 4-isopropylresorcinol, and 2-isopropylresorcinol. To identify the specific hydroxylated product of IPP, the degradation and growth of strain D-6 to the potential hydroxylated products were investigated separately. Strain D-6 completely degraded 1.04 mM 2-isopropylhydroquinone within 36 h (Fig. 2B), with its cell density increasing 24.0-fold from 3.0 × 10^6^ to 7.2 × 10^7^ CFU mL^−1^, while its cell density when cultured without 2-isopropylhydroquinone exhibited no significant change (Fig. 2B). These results indicated that 2-isopropylhydroquinone could serve as the sole growth substrate for strain D-6. However, after 35 h of incubation, strain D-6 could only degrade 0.34 mM 3-isopropylcatechol and 0.25 mM 4-isopropylresorcinol, with no increase in biomass (Figs. S5A and S5B). With prolonged incubation, neither 3-isopropylcatechol nor 4-isopropylresorcinol was completely degraded (data not shown). Additionally, strain D-6 was unable to degrade 2-isopropylresorcinol (Fig. S5 C). Moreover, the total cell density increment of strain D-6 cultured with 1.04 mM IPP was the same as that when cultured with equimolar 2-isopropylhydroquinone (Fig. 2). These results further showed that the hydroxylated product of IPP by strain D-6 was 2-isopropylhydroquinone, and not 3-isopropylcatechol as previously stated (22).

#### Disruption and complementation of *ippA1* in strain D-6

To investigate the role of *ippA1* in the conversion of IPP in strain D-6, an *ippA1* knockout strain D-6*∆ippA1* was constructed using a double-crossover event. Strain D-6*∆ippA1* lost its IPP degradation capability, while the complementary strain, D-6*∆ippA1/ippA1*, restored the ability to degrade IPP (Fig. S6). These results demonstrate that *ippA1* is required for IPP hydroxylation.

### 2-Isopropylhydroquinone degradation pathway in strain D-6

Strain D-6 initially hydrolyzes IPC to IPP using the hydrolase IpcH, followed by the hydroxylation of IPP to 2-isopropylhydroquinone by the two-component monooxygenase IppA1A2. To further investigate the IPC degradation pathway, strain D-6 was inoculated into MSM supplemented with 1.04 mM 2-isopropylhydroquinone. Cultures were collected every 2 h during 2-isopropylhydroquinone degradation, and the metabolites in this degradation process were detected through MS analysis. Six compounds (compounds I–VI) were identified during 2-isopropylhydroquinone degradation (Figs. S7A through F), including 2-isopropylhydroquinone (compound I), 2-isopropyl-4-hydroxymuconic semialdehyde (compound II), 2-isopropyl-maleylacetate (compound III), 5-isopropyl-3-ketoadipate (compound IV), 2,3-dimethylbutanoic acid (compound V), and malonic acid (compound VI) (Figs. S7A through F). The degradation pathway of 2-isopropylhydroquinone in strain D-6 was then speculated: initially, the strain catalyzes the ring cleavage of 2-isopropylhydroquinone to 2-isopropyl-4-hydroxymuconic semialdehyde, which is then dehydrogenated to 2-isopropyl-maleylacetate. Subsequently, 2-isopropyl-maleylacetate is reduced to yield 5-isopropyl-3-ketoadipate, which is further broken down into 2,3-dimethylbutanoic acid and malonic acid, and ultimately enters the tricarboxylic acid (TCA) cycle (Fig. S7G).

### IppB catalyzes 2-isopropylhydroquinone ring cleavage to generate 2-isopropyl-4-hydroxymuconic semialdehyde

#### Expression and purification of IppB

Within the *ipp* gene cluster, *orf2563* (5.95 log_2_ fold change, named *ippB*), located upstream of *ippA1A2*, was predicted to encode a dioxygenase responsible for catalyzing 2-isopropylhydroquinone ring cleavage (Table 1). IppB Exhibit 831% identity to biphenyl-2,3-diol 1,2-dioxygenase BphC2 from *Rhodococcus globerulus* P6 (31). To identify the function of *ippB*, it was expressed in *E. coli* BL21 (DE3). Subsequently, IppB was purified and subjected to SDS-PAGE analysis. Purified IppB appeared as a single band on SDS-PAGE, with a molecular mass of 23.4 kDa matching its theoretical molecular mass (Fig. S1C).

#### Characterization of the dioxygenase IppB

**Fig 3:**
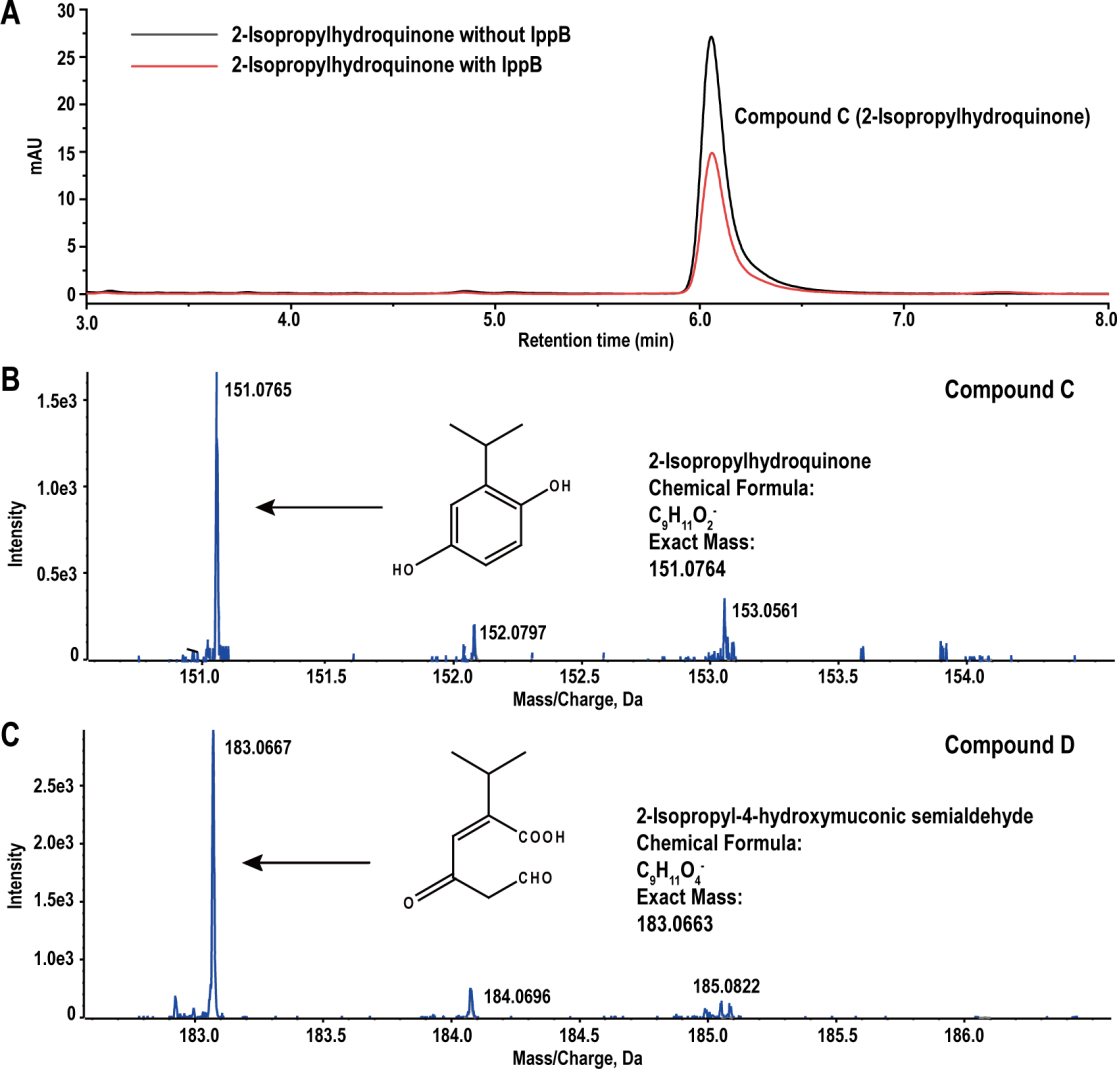
Identification of degradation product of 2-isopropylhydroquinone by IppB. (**A**) HPLC analysis of the degradation of 2-isopropylhydroquinone by IppB. (**B**) MS analysis of compound C (*m/z* 151.0765 [M-H]^−^), which was identified as 2-isopropylhydroquinone (C_9_H_11_O_2_^−^, *m/z* 151.0764) with a 0.3 ppm error. (**C**) MS analysis of compound D (*m/z* 183.0667 [M-H]^−^), which was identified as 2-isopropyl-4-hydroxymuconic semialdehyde (C_9_H_11_O_4_^−^, *m/z* 183.0663)..

The apparent enzyme kinetics of IppB for 2-isopropylhydroquinone, including *K*_m_, *k*_cat_, and *k*_cat_/*K*_m_, were 29.07 ± 1.54 µM, 147.65 ± 1.92 s^−1^, and 5.08 s^−1^ µM^−1^, respectively (Fig. S8). Notably, the substrate 2-isopropylhydroquinone with a retention time of 6.05 min decreased in the IppB enzymatic reaction mixture, while no product was detected via HPLC analysis (Fig. 3A). MS analysis was carried out to further identify the products, and two compounds were detected. The predominant deprotonated molecular ion of compound C was *m/z* 151.0765 [M-H]^−^, identified as 2-isopropylhydroquinone (C_9_H_11_O_2_^−^, *m/z* 151.0764) with a 0.3 ppm error (Fig. 3B). The molecular ion peak [M-H]^−^ of compound D was *m/z* 183.0667, corresponding to the deprotonated derivative of 2-isopropyl-4-hydroxymuconic semialdehyde (C_9_H_11_O_4_^−^, *m/z* 183.0663) with a 3.1 ppm error (Fig. 3C). These results suggested that IppB catalyzes 2-isopropylhydroquinone ring cleavage to produce 2-isopropyl-4-hydroxymuconic semialdehyde.

#### Identification of the conserved Fe(II) binding site HHE motif of IppB

IppB shared the highest identity with proteins belonging to the extradiol dioxygenase family, which are characterized by a highly conserved Fe(II) binding site HHE motif (32) (Fig. S9). Sequence alignment of IppB with BphC2 and BphC3 revealed that IppB also contains Fe(II) binding site residues His29, His92, and Glu140 (Fig. S10). To assess their roles in the function of IppB, these residues were individually replaced by alanine to generate three variants (IppB-H29A, IppB-H92A, and IppB-E140A) (Fig. S1C). Enzymatic assays showed that all variants lost their activity against 2-isopropylhydroquinone (Table S2), confirming that IppB is an extradiol dioxygenase containing a highly conserved Fe(II) binding site HHE motif.

#### Disruption and functional complementation of *ippB* in strain D-6

The *ippB* gene was knocked out using a double-crossover event to verify whether it is the only gene involved in 2-isopropylhydroquinone ring cleavage in strain D-6. The mutant strain D-6*ΔippB* lost the ability to convert 2-isopropylhydroquinone, while the complementary strain D-6*ΔippB/ippB* regained its degradation ability (Fig. S11A). In addition, strain D-6*ΔippB* accumulated an equimolar amount of 2-isopropylhydroquinone during IPP degradation, and the accumulated 2-isopropylhydroquinone was not further degraded (Fig. S11B). Moreover, strain D-6*ΔippB* also could not grow on IPP (Fig. S11B). These results indicated that *ippB* was responsible for cleaving the aromatic ring of 2-isopropylhydroquinone to 2-isopropyl-4-hydroxymuconic semialdehyde in strain D-6.

## DISCUSSION

**Fig 4:**
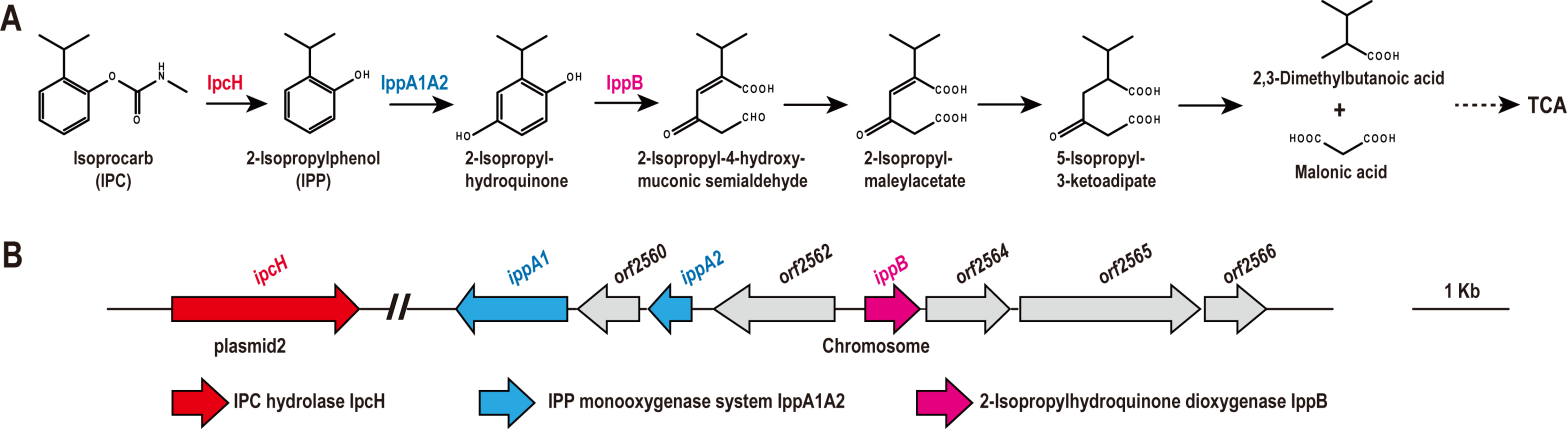
The proposed metabolic pathway of IPC and corresponding enzymes in strain D-6. (**A**) Proposed degradation pathway of IPC in strain D-6. (**B**) Genomic context of IPC degradation genes in strain D-6. The gene *ipcH* is located on plasmid2, and the *ipp* gene cluster is located on the chromosome.

To date, several IPC-degrading strains have been isolated from the genera *Streptococcus*, *Lactobacillus*, *Bacillus*, *Sphingobium*, *Acinetobacter*, and *Rhodococcus* (18–22). Among them, *Rhodococcus* sp. D-6, isolated in our lab, is the only strain capable of completely degrading IPC and utilizing it as the sole growth substrate (22). IPC is initially hydrolyzed to IPP by the hydrolase IpcH in strain D-6 (Fig. 4) (22). IPP is toxic and can pose risks to different organisms (22, 25–27). To date, the metabolic pathway of IPP has only been studied in *Pseudomonas* sp. strain HBP1 Prp, and IPP degradation genes remain uninvestigated.

Strain HBP1 Prp initiates the process by hydroxylating IPP to form 3-isopropylcatechol. 3-Isopropylcatechol subsequently undergoes ring cleavage and hydrolysis to produce isobutyric acid and 2-hydroxypent-2,4-dienoic acid, which enter the TCA cycle, providing the sole growth substrate for strain HBP1 Prp (28). In the present study, strain D-6 initially catalyzed IPP to generate 2-isopropylhydroquinone, which undergoes a series of steps involving dioxygenase-mediated ring cleavage, dehydrogenation, reduction, and hydrolysis to produce 2,3-dimethylbutanoic acid and malonic acid, and ultimately entered the TCA cycle to serve as growth substrates for strain D-6 (Fig. 4). The different IPP degradation pathways in strains D-6 and HBP1 Prp may be attributed to differences in the enzymes responsible for IPP degradation. A similar phenomenon has also been reported in previous studies. For instance, 4-nitrophenol is degraded via 1,2,4-benzenetriol by *Arthrobacter* sp. strain JS443 and *Rhodococcus opacus* SAO101, while it is degraded via the hydroquinone pathway by *Pseudomonas putida* DLL-E4 and *Burkholderia* sp. SJ98 (33–37).

The *ipp* gene cluster responsible for IPP catabolism was identified through transcriptomic analyses. Within the cluster, IppA1A2 was identified as the initial two-component monooxygenase involved in IPP catabolism. Phylogenetic analysis of IppA1 and related proteins showed that it clusters with group D flavin-dependent monooxygenases, forming a subclade with HsaA (exhibiting the highest similarity of 31.51% to IppA1), MpdA (27.46% similarity to IppA1), and C_2_-HPAH (24.67% similarity to IppA1) (Fig. S12A) (30, 38, 39). Further analysis of the IppA1 amino sequence revealed an acyl-coenzyme A (acyl-CoA) dehydrogenase fold in its C-terminal domain (Pro^239^-Asp^365^), which was characteristic of group D flavin-dependent monooxygenases (Fig. S13) (40). Group D flavin-dependent monooxygenases need a reductase partner protein for the delivery of reduced flavin to enable their catalytic function. This suggests that IppA1 also requires a reductase component to perform its catalytic activity. *ippA2*, located upstream of *ippA1*, encodes a flavin reductase that was confirmed to function as the reductase component of the IPP monooxygenase system (Fig. S12B). IppA2 showed 31.29%, 38.41%, and 30.46% identity with HsaB, MpdB, and C_1_-HPAH, respectively. Notably, HsaAB, MpdAB, and HPAH C_1_-C_2_ systems are all two-component flavin-dependent monooxygenases, responsible for catalyzing the hydroxylation of 3-hydroxy-9,10-secoandrosta-1,3,5(10)-triene-9,17-dione, 2,6-dimethylphenol, and *p*-hydroxyphenylacetate, respectively (30, 38, 39).

IppA1A2 catalyzes the hydroxylation of IPP at the *para* position of its hydroxyl group to generate 2-isopropylhydroquinone. This reaction differs from that of the partially purified monooxygenase in strain HBP1 Prp, which hydroxylates IPP at the *ortho* position, producing 3-isopropylcatechol (28). Due to the partial purification of the monooxygenase in strain HBP1 Prp, it remains unclear whether this monooxygenase is a single-component or two-component system that requires a separate reductase component. Additionally, the DNA and amino acid sequences encoding this monooxygenase remain unidentified. Consequently, it is not possible to compare the amino acid sequence similarity between IppA1 and this monooxygenase or to evaluate their respective catalytic efficiencies toward IPP.

IppB is a dioxygenase responsible for catalyzing the ring cleavage of 2-isopropylhydroquinone (Fig. 3). Ring-cleavage dioxygenases are typically classified into two groups based on their mode of aromatic ring cleavage: intradiol dioxygenases cleave the carbon-carbon (C-C) bond between the phenolic hydroxyl groups, while extradiol dioxygenases cleave the bond between a hydroxylated carbon and an adjacent non-hydroxylated carbon (41–43). Enzymatic characterization revealed that IppB specifically cleaves the C-C bond adjacent to the phenolic hydroxyl groups of 2-isopropylhydroquinone, generating 2-isopropyl-4-hydroxymuconic semialdehyde (Fig. 3). This cleavage mode is consistent with the extradiol-type cleavage. Further phylogenetic analysis showed that IppB clustered within the extradiol dioxygenase family, forming a subclade with BphC2 from *R. globerulus* P6 (exhibiting the highest similarity of 83.68% to IppB) and BphC3 from strain P6 (60.11% identity to IppB) (Fig. S9) (31). Both BphC2 and BphC3 function as extradiol dioxygenases involved in catalyzing the *meta* cleavage of 2,3-dihydroxybiphenyl to produce 2-hydroxy-6-oxo-6-phenylhexa-2,4-dienoic acid (31, 44). From this, it can be concluded that IppB is an extradiol dioxygenase. The extradiol dioxygenase family is efficient in catalyzing the ring cleavage of various phenolic compounds, such as the conversion of caffeic acid into *iso*-arabidopic acid through 2,3-extradiol cleavage by dioxygenase AtLigB (45), the cleavage of 3-methylcatechol to form 2-hydroxy-6-oxo-2,4-heptadienoate by 3-methylcatechol 2,3-dioxygenase TodE (46), and the transformation of catechol into 2-hydroxymuconate-6-semialdehyde by catechol 2,3-dioxygenase BztE (47).

IPC and its initial degradation product IPP can cause environmental pollution and pose potential toxicological risks to non-target organisms (22–27, 48–52). Notably, our previous results showed that IPP exhibits higher toxicity toward Gram-negative strain *E. coli* DH5α, Gram-positive strain *B. cereus* 168, yeast strain *P. pastoris* GS115, and algae *C. ellipsoidea* compared to its parent substrate IPC (22). Consequently, achieving the complete degradation of IPC and IPP is critical for mitigating environmental contamination and its associated hazards. Strain D-6 has demonstrated the capacity to completely mineralize IPC and IPP, indicating its potential in remediating environments contaminated with these compounds.

## MATERIALS AND METHODS

### Chemicals and media

IPP (98.0%) and 2-isopropylhydroquinone (99.5%) were obtained from Macklin Biochemical Co., Ltd. (Shanghai, China) and YuanYe Bio-Technology Co., Ltd. (Shanghai, China), respectively. Stock solutions of IPP (5.0 g L^−1^) and 2-isopropylhydroquinone (5.0 g L^−1^) were prepared using methanol as the solvent, which cannot serve as a growth substrate for strain D-6 (data not shown). The reduced nicotinamide adenine dinucleotide phosphate (NADPH), reduced nicotinamide adenine dinucleotide (NADH), flavin mononucleotide (FMN), and flavin adenine dinucleotide (FAD) were obtained from Aladdin Reagent Co., Ltd. (Shanghai, China). Luria-Bertani (LB, pH 7.0) medium comprised 10 g L^−1^ tryptone, 10.0 g L^−1^ NaCl, and 5.0 g L^−1^ yeast extract. MSM (pH 7.0) comprised 1.5 g L^−1^ K_2_HPO_4_, 1.0 g L^−1^ NH_4_NO_3_, 1.0 g L^−1^ NaCl, 0.5 g L^−1^ KH_2_PO_4_, and 0.2 g L^−1^ MgSO_4_·7H_2_O. The growth substrates IPP and 2-isopropylhydroquinone were added as required.

### Strains, plasmids, and culture conditions

All bacterial strains and plasmids utilized in this study are listed in Table S3, and the primers are listed in Table S4. *E. coli* strains were cultured in LB medium at 37°C. *Rhodococcus* sp. D-6 and its derivatives were grown at 30°C in LB medium. Antibiotics were supplemented according to Table S4. Streptomycin (Str) and kanamycin (Km) were used at a final concentration of 25 and 50 µg mL^−1^, respectively.

### Transcriptome analysis

Strain D-6 was cultured in LB medium until reaching the mid-log phase. Subsequently, the cells were harvested by centrifugation at 6,000 × *g* for 5 min and washed three times with MSM. The washed cells were then suspended in MSM supplemented with 1.04 mM IPP to a final optical density at 600 nm (OD_600_) of approximately 0.2. MSM containing strain D-6 (final OD_600_ of 0.2) and 1.04 mM glucose was used as a control. When approximately 50.0% of IPP had degraded, the cells were collected for transcriptome sequencing. Both IPP and glucose treatments were conducted in triplicate for the transcriptomic analysis. Transcriptome sequencing was performed by Shanghai Biozeron Technology Co., Ltd.

### Gene expression and purification

The *ippA1* gene was amplified from the genomic DNA of strain D-6 using primers *ippA1*-F and *ippA1*-R (Table S4). The *ippA1* gene fragment was digested with *BamH*I and *EcoR*I and subsequently ligated into the *BamH*I-*EcoR*I sites of the pET-28a vector to create the recombinant plasmid pET-*ippA1*. pET-*ippA1* was sequenced to verify its accuracy and was transformed into *E. coli* BL21 (DE3) for *ippA1* expression. *E. coli* carrying pET-*ippA1* was cultured in LB broth at 37°C. After OD_600_ reached 0.6, 0.1 mM isopropyl-*β*-d-thigalactopyranoside (IPTG) was added to the medium and the cells were induced for 10 h at 16°C. The cells were harvested and resuspended in 15 mL Tris-HCl buffer and lysed via sonication. Each ultrasonic cycle consisted of 1 s sonication at 50% intensity followed by a 2 s pause at 4°C. Sonication was carried out for a total of 20 min. Subsequently, the crude extract was collected by centrifugation at 4°C and 12,000 × *g* for 30 min.

To purify the protein IppA1, the supernatant was applied to Ni^2+^-nitrilotriacetic acid-agarose chromatography columns and dialyzed in 20 mM Tris-HCl buffer (pH 7.4) overnight to remove the imidazole and Ni^2+^. The purified IppA1 was analyzed by sodium dodecyl sulfate-polyacrylamide gel electrophoresis (SDS-PAGE), and its concentration was measured using the Bradford method (53). *ippA2*, *ippB,* and their mutants were expressed and purified similarly.

### Enzyme assays

All enzyme reactions were performed in 1 mL Tris-HCl buffer (20 mM, pH 7.4) at 30°C. The NAD(P)H-oxidizing activity of the reductase IppA2 was assessed by measuring the continuous consumption at 340 nm using a UV scanning method. The kinetic assays of IppA2 for NADH were performed in Tris-HCl buffer containing 0.4 µM IppA2, various concentrations of NADH (0–300 µM), and 10 µM FAD. The kinetic assays of IppA2 for FAD were conducted in Tris-HCl buffer containing 0.4 µM IppA2, 250 µM NADH, and different concentrations of FAD (0–20 µM). The reaction was initiated by adding NAD(P)H.

To evaluate the molar ratio of IppA1 to IppA2, a reaction solution containing 220 µM IPP, 250 µM NADH, 10 µM FAD, 1.2 µM IppA1, and IppA2 at various concentrations ranging from 0 to 1.50 µM was used. The standard enzyme reaction for IppA1A2 contained 220 µM IPP, 250 µM NADH, 10 µM FAD, 1.2 µM IppA1, and 0.4 µM IppA2. To investigate the kinetics of IppA1A2 against IPP, different concentrations of IPP (0–810 µM) were used in the presence of 250 µM NADH, 10 µM FAD, 1.2 µM IppA1, and 0.4 µM IppA2. The apparent values of *K*_m_ and *k*_cat_ of IppA1 for IPP were determined through nonlinear regression fitting of the Michaelis-Menten kinetic equation to the experimental data.

The standard enzyme reaction system for IppB was 200 µM 2-isopropylhydroquinone, 1.0 µM IppB, and 10 µM Fe^2+^. The kinetics of IppB was explored by using different concentrations of 2-isopropylhydroquinone (0–460 µM) with 1.0 µM IppB and 10 µM Fe^2+^. The calculation methods for the apparent values of *K*_m_ and *k*_cat_ of IppB against 2-isopropylhydroquinone were similar to those for IppA1 against IPP.

### Site-directed mutagenesis

Point mutations in *ippB* were constructed by overlap PCR as described by Zhu et al. (54). The forward and reverse flanking primers were primer *ippB*-F and *ippB*-R, respectively. The internal primer pairs H29A-F/H29A-R, H92A-F/H92A-R, and E140A-F/E140A-R are shown in Table S4. PCR products were gel-purified and then cloned into the *Bam*HI and *Eco*RI sites of the pET-28a(+) plasmid; the proteins were purified, and their activity against 2-isopropylhydroquinone was assessed according to the method described above.

### Gene disruption and genetic complementation

Two DNA fragments corresponding to the 1,300 bp upstream and downstream flanking regions of the *ippA1* gene were amplified from strain D-6 to disrupt *ippA1* through a double-crossover procedure. Initially, the two fragments were combined using an overlap extension PCR and subsequently ligated into pK18*mobsacB* (55) to generate pK18-*ippA1*, which was then introduced into strain D-6 through electroporation. The single-crossover mutant was selected on LB agar containing 25 µg mL^−1^ streptomycin and 50 µg mL^−1^ kanamycin. After culturing the verified single-crossover mutant until the OD_600_ approached approximately 0.2, it was serially diluted on LB agar containing 25 µg mL^−1^ streptomycin and 20% sucrose to isolate the double-crossover mutant. Both the single and double-crossover mutants were confirmed through PCR and DNA sequencing, with the resulting mutant designated D-6*∆ippA1*. A similar approach was used to generate the *ippB* knockout mutant D-6*∆ippB*.

To generate the *ippA1*-complementary strain D-6*∆ippA1/ippA1*, the *ippA1* gene was amplified from the genomic DNA of strain D-6 using primers listed in Table S4. The resulting DNA fragment was then ligated into pRESQ (56) to create pRE-*ippA1*. Subsequently, pRE-*ippA1* was introduced into strain D-6*∆ippA1* through electroporation for complementation. The complementary strain D-6*∆ippB/ippB* was generated using the same method.

The ability of D-6*∆ippA1*, D-6*∆ippA1/ippA1*, and D-6*∆ippB* to degrade IPP was assessed in MSM supplemented with 1.04 mM IPP. The ability of D-6*∆ippB* and D-6*∆ippB/ippB* to degrade 2-isopropylhydroquinone was assessed in MSM supplemented with 1.04 mM 2-isopropylhydroquinone.

### Degradation experiments by strain D-6

Cells of strain D-6 were grown in LB medium, harvested by centrifugation (5,000 × *g* for 5 min) at mid-log phase, and washed three times with MSM. The cells (3.0 × 10^6^ CFU mL^−1^) were suspended in 100 mL sterilized MSM in the presence of 1.04 mM IPP. Cultures were collected periodically from each treatment to determine the growth of strain D-6 and measure the residual IPP concentration by HPLC as described in the Analytical Methods. Cell growth was determined with the colony counting method. The degradation and growth experiments were carried out for 2-isopropylhydroquinone, 3-isopropylcatechol, 4-isopropylresorcinol, and 2-isopropylresorcinol at 1.04 mM, following a comparable approach to the IPP experiments. MSM without strain D-6 under the same conditions was set as a control. All the degradation experiments were triplicated. The metabolites of 2-isopropylhydroquinone degradation by strain D-6 were identified using mass spectrometer (MS) as described in the Analytical Methods.

### Analytical methods

The degradation culture and enzyme reaction samples were fully mixed with an equal volume of methanol, followed by centrifugation (12,000 × *g* for 5 min). Subsequently, the supernatants were filtered through 0.22 μm-pore filters. The concentrations of IPP, 2-isopropylhydroquinone, 3-isopropylcatechol, 4-isopropylresorcinol, and 2-isopropylresorcinol were detected by HPLC (Thermo Fisher Scientific, Massachusetts) with a C_18_ reverse-phase column (4.6 by 250 mm, 5 µm). The mobile phase consisted of methanol-water-acetic acid (50:49:1, vol/vol/vol) at a flow rate of 1.0 mL min^−1^. The column elution was monitored by measuring the absorbance at 215 nm. The injection volume was 20 µL, and the column temperature was maintained at 40°C. IPP, 2-isopropylhydroquinone, and its metabolites were identified using an AB SCIEX Triple Time-of-Flight 5600 Plus high-resolution MS equipped with a Turbo V probe.

## Data Availability

The genome sequence of *Rhodococcus* sp. D-6 was deposited into the GenBank database under the accession numbers CP132970 to CP132972.

## SUPPLEMENTAL MATERIAL

Supplemental material aem.00995-25-s0001.pdf Tables S1 to S4; Fig. S1 to S13.

Tables S5 to S10 aem.00995-25-s0002.xlsx Gene expression data.

ASM does not own the copyrights to Supplemental Material that may be linked to, or accessed through, an article. The authors have granted ASM a non-exclusive, world-wide license to publish the Supplemental Material files. Please contact the corresponding author directly for reuse.
