# Supplementary material for: Identification of a novel ipp gene cluster responsible for 2-isopropylphenol degradation in strain Rhodococcus sp. D-6
Source: Appl Environ Microbiol. 2025 Aug 14;91(9):e00995-25. doi: 10.1128/aem.00995-25 (PMC12442392; doi:10.1128/aem.00995-25)
Supplement: Supplemental material — Tables S1 to S4; Fig. S1 to S13. [file aem.00995-25-s0001.pdf]

Identification of a Novel *ipp* Gene Cluster Responsible for 2-Isopropylphenol Degradation in strain *Rhodococcus* sp. D-6

Qian Zhu, [a] Kangning Wei, [a] Kaihua Pan, [a] Gang Hu, [b] Weihao Zhu, [a] Yanni Huang, [a] Changchang Wang, [a] Qian Li, [a] Mingliang Zhang, [a] Jiguo Qiu, [a] Qing Hong, [a,#]

[a] Department of Microbiology, College of Life Sciences, Nanjing Agricultural University, Key Laboratory of Agricultural and Environmental Microbiology, Ministry of Agriculture and Rural Affairs, Nanjing 210095, China.

[b] Modern Agricultural Analysis and Testing Center, Nanjing Agricultural University, Nanjing, Jiangsu 210095, PR China.

[#] Author for correspondence: Qing Hong

E-mail address: hongqing@njau.edu.cn.

Tel: +86-25-84396685; Fax: +86-25-84395326

RUNNING TITLE: The novel 2-isopropylphenol degradation gene cluster.

KEYWORDS: *Rhodococcus* sp. D-6, biodegradation, isoprocarb, 2-isopropylphenol monooxygenase IppA1A2, 2-isopropylhydroquinone dioxygenase IppB

Table S1 Summary of gene expression in strain D-6 cultured with glucose and IPP

Sample name	Glucose treatments	IPP treatments
Clean reads	16,090,993	15,938,567
Clean rate (%)	98.66	98.09
Genome mapped reads	14,739,488	15,279,075
Genome mapped rate (%)	91.55	95.85
Expressed genes	5,097	5,101
Differentially expressed genes	4,183	
Up-regulated genes	3,735	
Down-regulate genes	448	

Table S2 Comparison of the relative activities of IppB and its variants.

Enzymes	Relative activity [a] (%)
IppB	100 ± 1.3
IppB-H29A	ND
IppB-H92A	ND
IppB-E140A	ND

[a] Results are the average of triplicate measurements ± standard deviation. The relative activity was calculated by assuming the IppB activity observed was 100%.

Table S3 Strains and plasmids used in this study

Strain or plasmid	Characteristic(s)[a]	Source or reference(s)
E. coli strains		
DH5α	F^-*recA1 endA1 thi-1 supE44 relA1 deoRΔ(lacZYA-argF)U169 80dlacZΔM15*	TaKaRa
BL21 (DE3)	F^-*ompT hsdS*(r_B^- m_B^-) *gal dcm lacY1* (DE3)	TaKaRa
Rhodococcus sp. strains		
D-6	Wild type, able to degrade IPC and IPP; Str^r	This study
D-6Δ*ippA1*	*ippA1*-disrupted mutant from stain D-6; Str^r	This study
D-6Δ*ippA1/ippA1*	D-6*ΔippA1* harboring pRE-*ippA1*; Str^r	This study
D-6Δ*ippB*	*ippB*-disrupted mutant from stain D-6; Str^r	This study
D-6Δ*ippB/ippB*	D-6*ΔippB* harboring pRE-*ippB*; Str^r	This study
Plasmids		
pK18*mobsacB*	Gene knockout vector; *ori*T, *sac*B, Km^r	[54]
pRESQ	Cloning vector; Km^r	[55]
pK18-*ippA1*	*ippA1* gene knockout vector containing upstream and downstream homologous regions of *ippA1*; Km^r	This study
pRE-*ippA1*	pRESQ harboring *ippA1*; Km^r	This study
pK18-*ippB*	*ippB* gene knockout vector containing upstream and downstream homologous regions of *ippB*; Km^r	This study
pRE-*ippB*	pRESQ harboring *ippB*; Km^r	This study
pET-28a (+)	Expression vector, Km^r	Novagen
pET-*ippA1*	pET-28a (+) harboring *ippA1*, Km^r	This study
pET-*ippA2*	pET-28a (+) harboring *ippA2*, Km^r	This study
pET-*ippB*	pET-28a (+) harboring *ippB*, Km^r	This study
pET-H29A	pET-28a (+) harboring *ippB*-H29A, Km^r	This study
pET-H92A	pET-28a (+) harboring *ippB*-H92A, Km^r	This study
pET-E140A	pET-28a (+) harboring *ippB*-E140A, Km^r	This study

[a] Str^r, Streptomycin resistant; Km^r, kanamycin resistant.

Table S4 Oligonucleotides used in this study

Primer	Sequence (5′→3′)	Purpose
ippA1-F	CAGCAAATGGGTCGCGGATCCATGGACATCACCACCGAACAACTG	Construction of plasmid pET-*ippA1*
ippA1-R	TTGTCGACGGAGCTCGAATTCTCAGAGCAGCGGGTCGGC	
ippA2-F	CAGCAAATGGGTCGCGGATCCTTGAGTTCGCTACAGGAAACTTTCA	Construction of plasmid pET-*ippA2*
ippA2-R	TTGTCGACGGAGCTCGAATTCTTACACCGTTTCGAGGGCG	
ippB-F	CAGCAAATGGGTCGCGGATCCATGCGCATGCCCCTGTGG	Construction of plasmid pET-*ippB*
ippB-R	TTGTCGACGGAGCTCGAATTCTCAGCTGGTCAGTGCGACC	
H29A-F	GCGGCTGGTCTGGAGCACAACTGCCGCGAACTTGGGGGTAGAGGT	Construction of plasmid pET-*ippB*-H29A
H29A-R	ACCTCTACCCCCAAGTTCGCGGCAGTTGTGCTCCAGACCAGCCGC	
H92A-F	GGTGTCGAAGGTGTAGGCCGATGCATGCATGCCGGCGGCGCCCGG	Construction of plasmid pET-*ippB*-H92A
H92A-R	CCGGGCGCCGCCGGCATGCATGCATCGGCCTACACCTTCGACACC	
E140A-F	AGCGAAGTTGTCGATCTGCAGTGCGACGAAGTTCCCGTCGGGGTC	Construction of plasmid pET-*ippB*-E140A
E140A-R	GACCCCGACGGGAACTTCGTCGCACTGCAGATCGACAACTTCGCT	
ippA1-upF	CTATGACATGATTACGAATTCGACACTTCCGTTGAGTTCGC	Construction of plasmid pK18-*ippA1*
ippA1-upR	CTCGAGTTGTCAGAGCAGCGGGTCCATGTTTTCCTCACTTCGC	
ippA1-downF	CCGCTGCTCTGACAACTC	Construction of plasmid pK18-*ippA1*
ippA1-downR	CAGGTCGACTCTAGAGGATCCGGTGTGCCAGAACTGACC	
ippA1-hbF	ACCGAGCTCAGATCTACTAGTATGGACATCACCACCGAACA	Construction of plasmid pRE-*ippA1*
ippA1-hbR	ACACTGGCGGCCGTTACTAGTGGTACACCGGCTCGAGTT	
ippB-upF	CTATGACATGATTACGAATTCAAGCTCGATCATCCGCGG	Construction of plasmid pK18-*ippB*
ippB-upR	GAGAACGAATCCTTCGAGACGCGAATGGGACACCGGTTG	
ippB-downF	CGTCTCGAAGGATTCGTTCTC	Construction of plasmid pK18-*ippB*
ippB-downR	CAGGTCGACTCTAGAGGATCCGGATACCAGTTCGTCGCGTCC	
ippB-hbF	ACCGAGCTCAGATCTACTAGTCCAGCCCCCTCCGATTCC	Construction of plasmid pRE-*ippB*
ippB-hbR	ACACTGGCGGCCGTTACTAGTATCGGGAAGAGTGTCGGAATC	

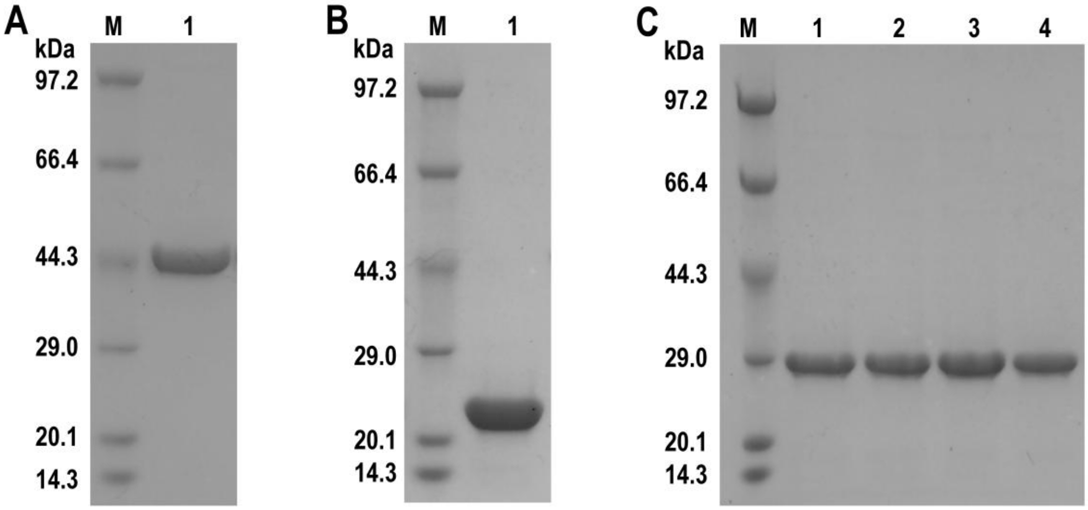

Fig. S1. SDS-PAGE analysis of the purified proteins. (A) SDS-PAGE of the purified IppA1. Lane M, protein marker; lane 1: the purified IppA1. (B) SDS-PAGE of the purified IppA2. Lane M, protein marker; lane 1: the purified IppA2. (C) Lane M, protein marker; lane 1: the purified IppB; lane 2: IppB-H29A; lane 3: IppB-H92A; lane 4: IppB-E140A.

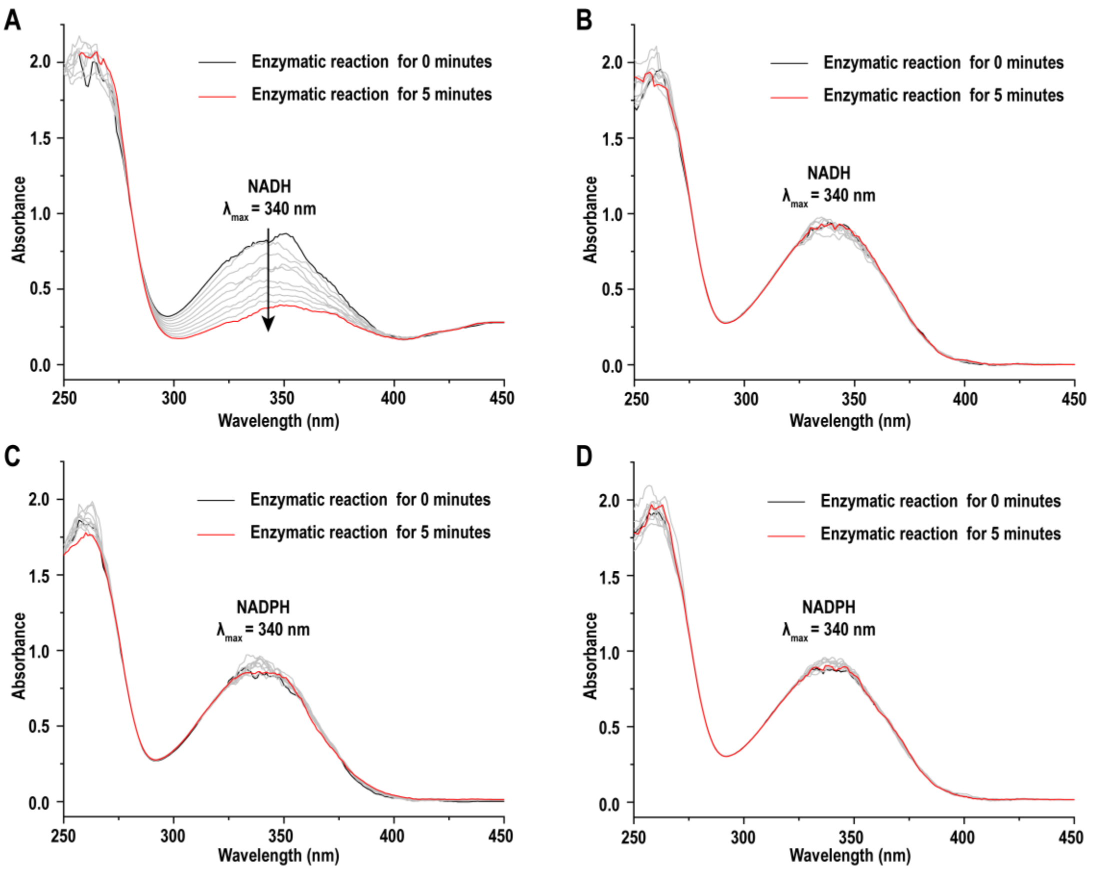

Fig. S2. The activity of reductase IppA2. (A) IppA2 mediated oxidation of NADH with FAD. (B) IppA2 mediated oxidation of NADH with FMN. (C) IppA2 mediated oxidation of NADPH with FAD. (D) IppA2 mediated oxidation of NADPH with FMN. Each reaction contained 0.4 μM IppA2, 250 μM NADH/NADPH, and 10 μM FAD/FMN. The UV spectra at 250 to 450 nm were monitored every 30 s. The reaction was started by the addition of NADH/NADPH. The arrows indicate the directions of spectral changes.

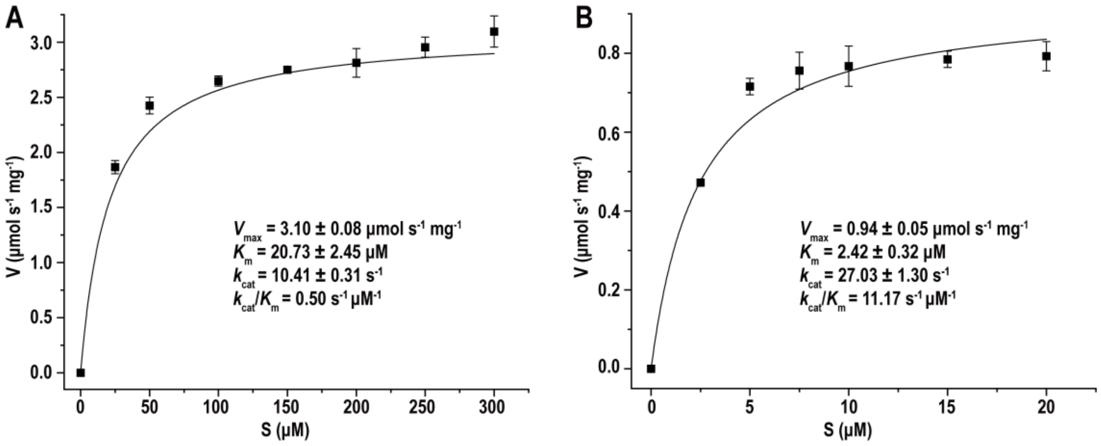

Fig. S3. Enzymatic characterization of IppA2 towards NDAH (A) or FAD (B). (A) NADH was used as the substrate, and FAD was used as the second substrate. (B) FAD was used as the substrate, and NADH was used as the second substrate.

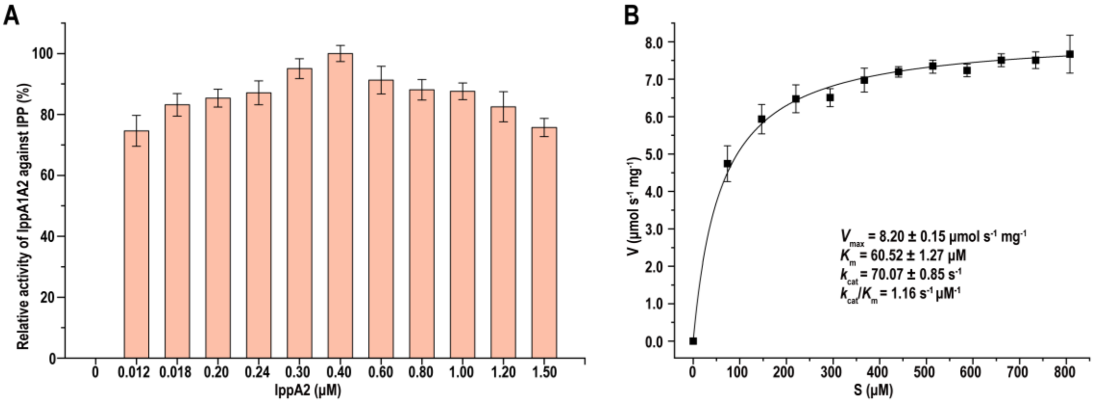

Fig. S4. Functional identification of the two-component flavin-dependent monooxygenase IppA1A2. (A) Relationship between the IPP hydroxylation activity and various concentrations of IppA2 while the concentration of IppA1 was kept constant. The concentration of IppA2 increased from 0 to 1.50 µM, while the IppA1 concentration was kept constant at 1.20 µM. The specific activity of IppA1 against IPP was defined as 100% when 0.4 µM IppA2 was added. (B) The kinetic curve of IppA1A2 with IPP as substrate. Error bars represent the standard errors for three replicates. Data were calculated from three independent replicates, and the error bars indicate standard deviations.

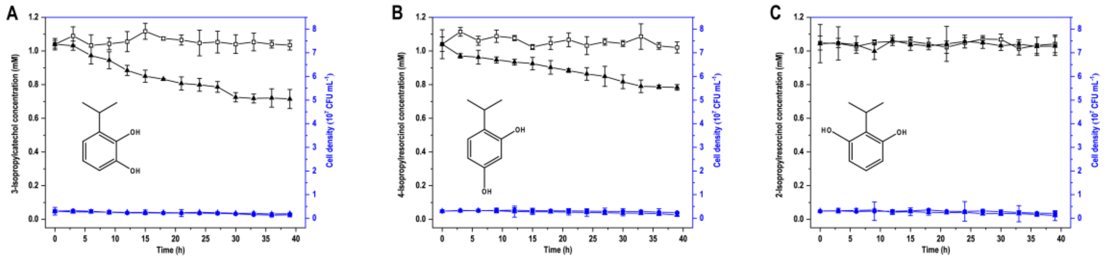

Fig. S5. Degradation and growth of strain D-6 on three substrates: 3-isopropylcatechol (A), 4-isopropylresorcinol (B) and 2-isopropylresorcinol (C). Figures A-C display the molecular structural formula of each substrate; □ indicates the substrate control; ▲ indicates the substrate with strain D-6; ● indicates the cell density of strain D-6 in the presence of the substrate; ▲ represents the cell density of strain D-6 in the absence the substrate. Cell growth was quantified using the colony counting method. Error bars indicate the standard errors derived from triplicate experiments.

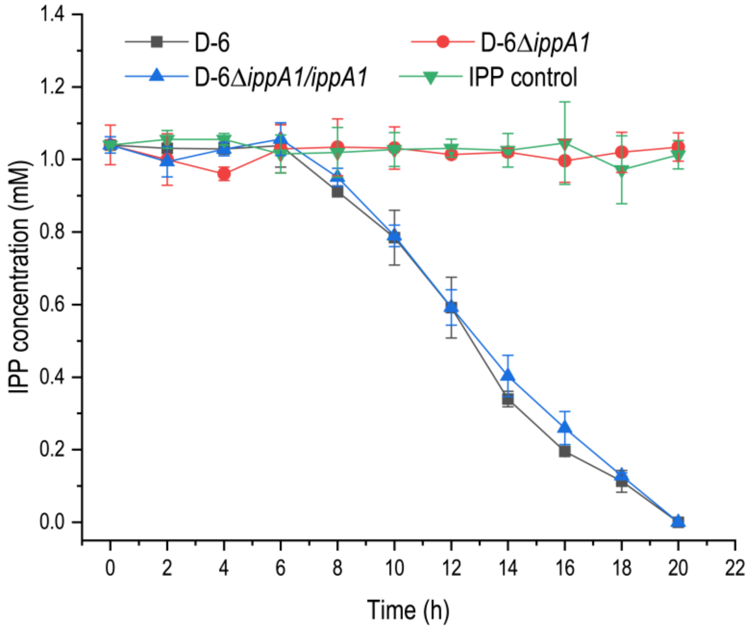

Fig. S6. Degradation of IPP by wild-type strain D-6, the *ippA1* knockout mutant strain D-6Δ*ippA1* and *ippA1*-complementary strain D-6Δ*ippA1/ippA1*.

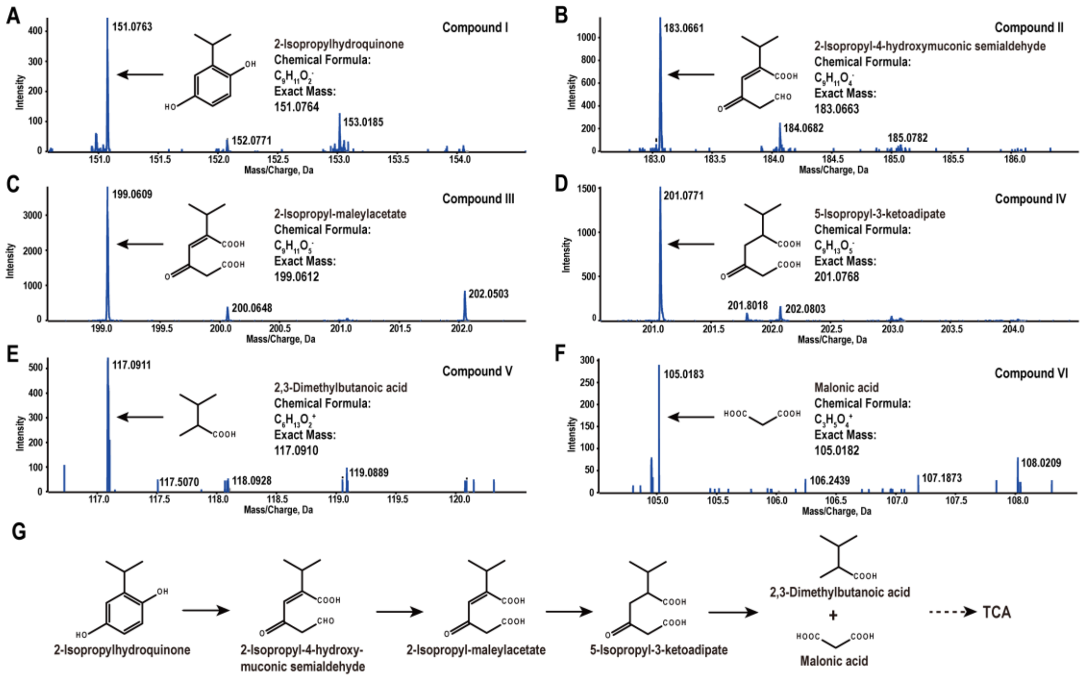

Fig. S7. Degradation pathway of 2-isopropylhydroquinone in strain D-6. (A) MS analysis of metabolites during the degradation of 2-isopropylhydroquinone by strain D-6. Compound I (*m/z* 151.0763) was identified as 2-isopropylhydroquinone ($C_9H_{11}O_2^-$, *m/z* 151.0764) with a -0.8 ppm error. (B) Compound II (*m/z* 183.0661) was identified as 2-isopropyl-4-hydroxymuconic semialdehyde ($C_9H_{11}O_4^-$, *m/z* 183.0663) with -0.8 ppm error. (C) Compound III (*m/z* 199.0609) was identified as 2-isopropyl-maleylacetate ($C_9H_{11}O_5^-$, *m/z* 199.0612) with -1.4 ppm error. (D) Compound IV (*m/z* 201.0771) was identified as 5-isopropyl-3-ketoadipate ($C_9H_{13}O_5^-$, *m/z* 201.0768) with 1.5 ppm error. (E) Compound V (*m/z* 117.0911) was identified as 2,3-dimethylbutanoic acid ($C_6H_{13}O_2^+$, *m/z* 117.0910) with 0.7 ppm error. (F) Compound VI (*m/z* 105.0183) was identified as malonic acid ($C_3H_5O_4^+$, *m/z* 105.0182) with 0.2 ppm error. (G) The proposed metabolic pathway of 2-isopropylhydroquinone degradation in strain D-6.

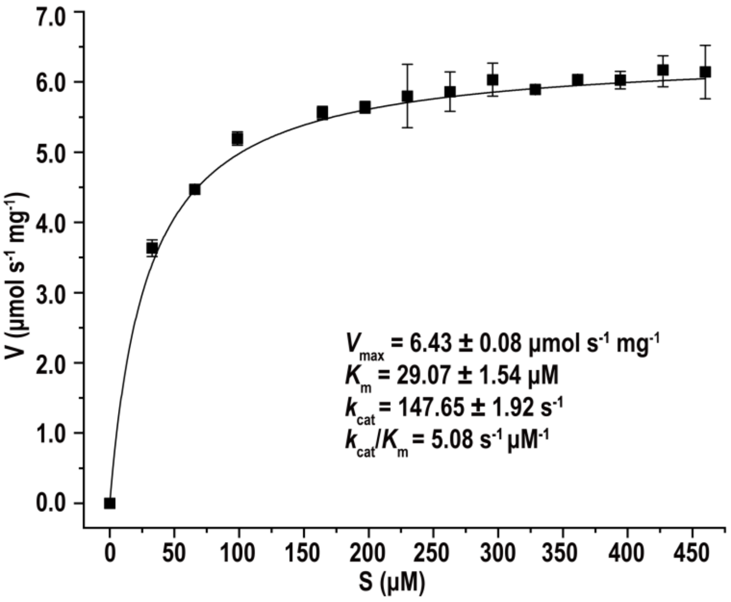

Fig. S8. The kinetic curve of IppB with 2-isopropylhydroquinone as substrate. Error bars represent the standard errors for three replicates.

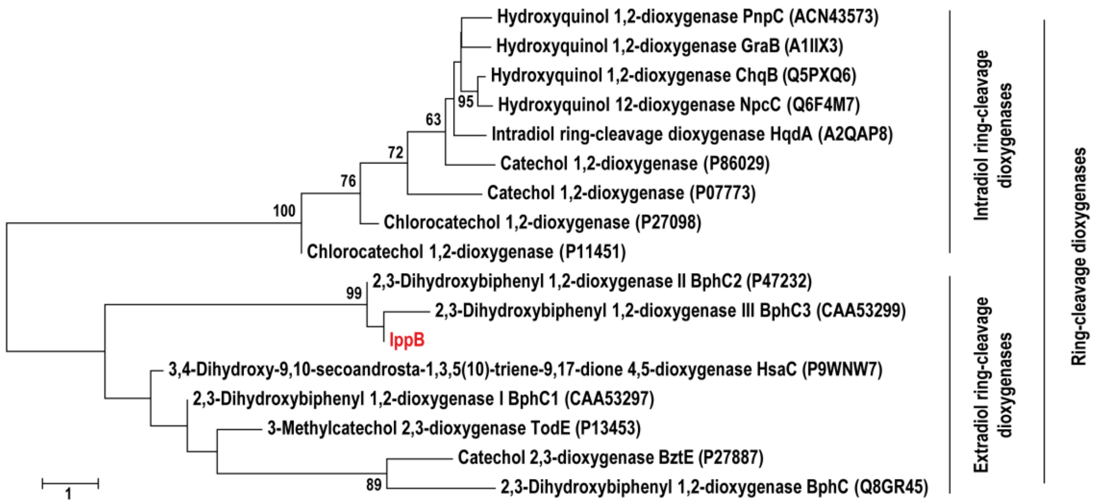

Fig. S9. Phylogenetic analysis of IppB and related proteins by neighbor-joining method. Bootstrap values (%) are indicated at the branch nodes, and the scale bar represents 1.0 substitutions per site. The accession numbers are in parentheses.

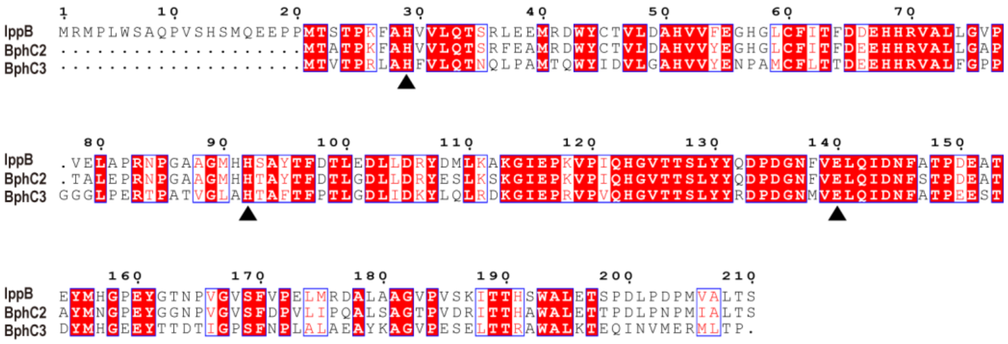

Fig. S10. Alignment of IppB with the extradiol ring-cleavage dioxygenase family, including BphC2 (P47232) and BphC3 (CAA53299). The alignment was generated using ClustalX. The conserved Fe(II) binding site (His29, His92 and Glu140) was indicated by black arrow.

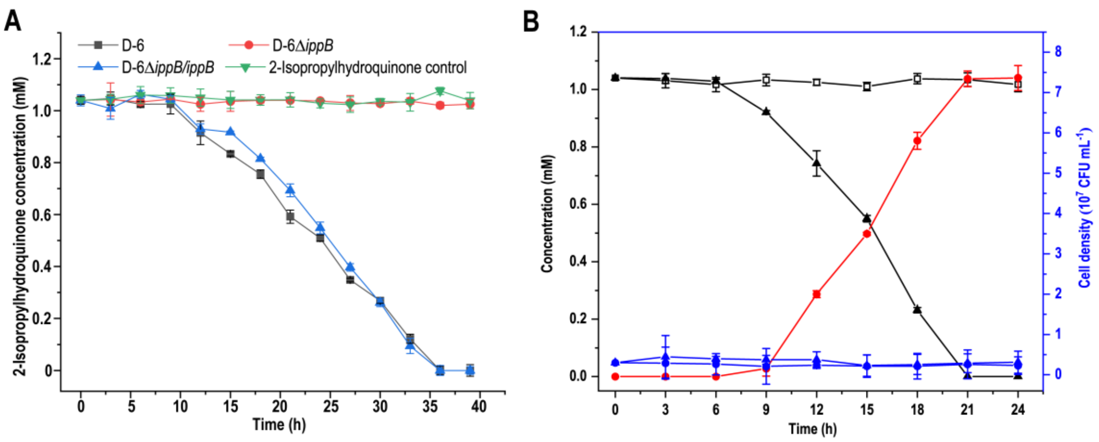

Fig. S11. (A) Degradation of 2-isopropylhydroquinone by wild-type strain D-6, the *ippB* knockout mutant strain D-6Δ*ippB* and *ippB*-complementary strain D-6Δ*ippB/ippB*. (B) Degradation and growth of strain D-6Δ*ippB* on IPP. □ represents the IPP control; ▲ represents IPP with strain D-6Δ*ippB*; ● represents 2-isopropylhydroquinone produced during IPP degradation by strain D-6Δ*ippB*; ● represents the cell density of strain D-6Δ*ippB* with IPP; ▲ represents the cell density of strain D-6Δ*ippB*.without IPP. Cell growth was determined by the colony counting method.

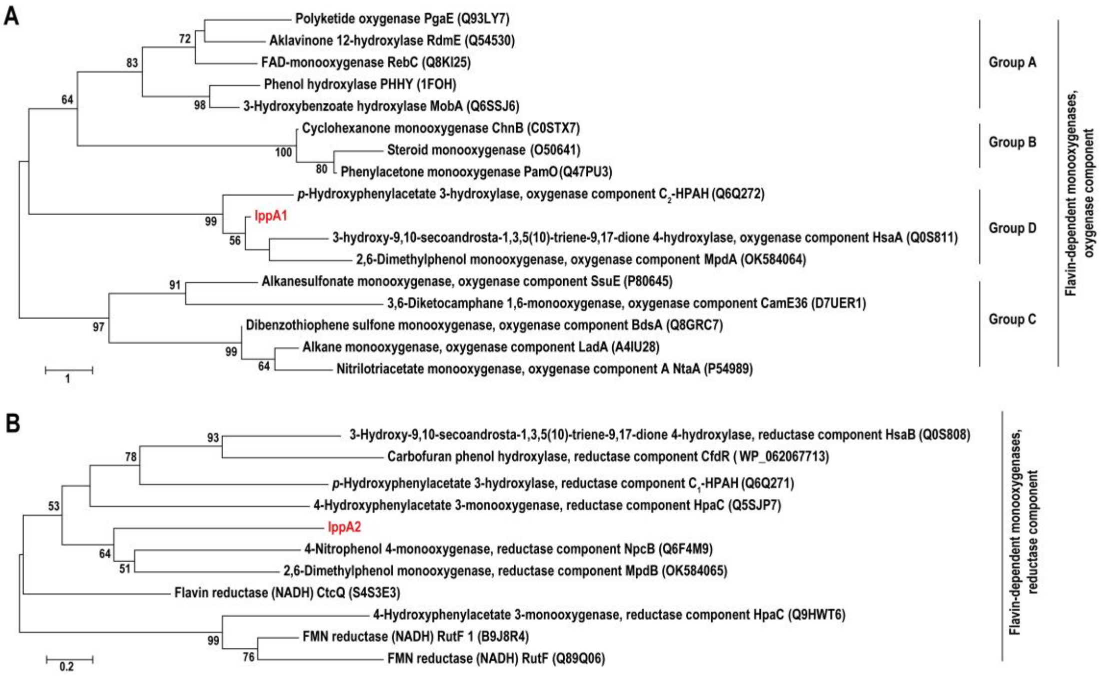

Fig. S12. Phylogenetic analysis of IppA1 and IppA2 (A) Phylogenetic tree was constructed based on the alignment of IppA1 with the oxygenase components of related flavin-dependent monooxygenase. The scale bar represents 1.0 substitutions per site. (B) Phylogenetic tree of IppA2 with the reductase components of related flavin-dependent monooxygenase. The scale bar represents 0.2 substitutions per site. Bootstrap values (%) are indicated at the branch nodes. The accession numbers are in parentheses.

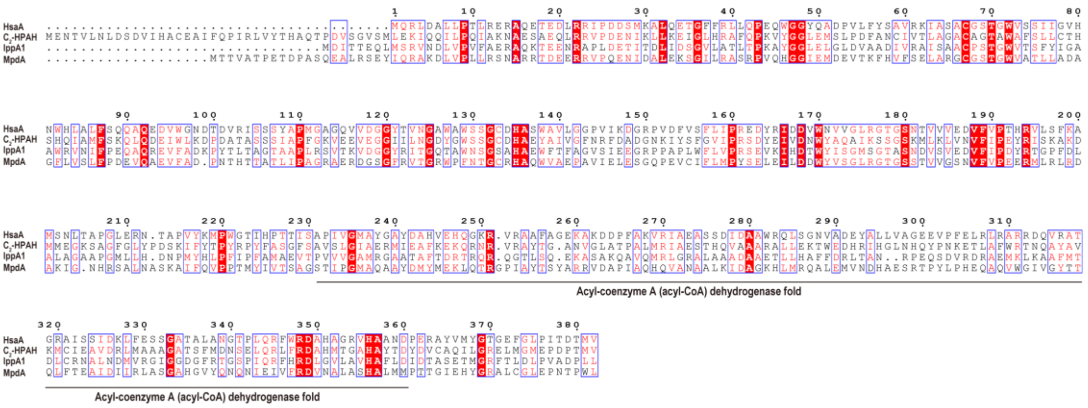

Fig. S13. Alignment of IppA1 with the oxygenase component of group D flavin-dependent monooxygenases, including HsaA (Q0S811), C_2-HPAH (Q6Q272) and MpdA (OK584064). The alignment was generated using ClustalX. The acyl-coenzyme A (acyl-CoA) dehydrogenase fold of the C-terminal domains is underlined.
